# Technical–tactical analysis of small combat games in male kickboxers: effects of varied number of opponents and area size

**DOI:** 10.1186/s13102-021-00391-0

**Published:** 2021-12-17

**Authors:** Ibrahim Ouergui, Slaheddine Delleli, Anissa Bouassida, Ezdine Bouhlel, Helmi Chaabene, Luca Paolo Ardigò, Emerson Franchini

**Affiliations:** 1 High Institute of Sport and Physical Education of Kef, Kef, University of Jendouba, Jendouba, Tunisia; 2 Laboratory of Cardio-Circulatory, Respiratory, Metabolic and Hormonal Adaptations to Muscular Exercise, Faculty of Medicine Ibn El Jazzar, University of Sousse, Sousse, Tunisia; 3 Department of Sports and Health Sciences, Faculty of Human Sciences, University of Potsdam, 14469 Potsdam, Germany; 4 Department of Neurosciences, Biomedicine and Movement Sciences, School of Exercise and Sport Science, University of Verona, Verona, Italy; 5 Martial Arts and Combat Sports Research Group, School of Physical Education and Sport, University of São Paulo, São Paulo, Brazil

**Keywords:** Martial arts, Time-motion analysis, Punch, Kick, Defensive actions

## Abstract

**Background:**

To handle the competition demands, sparring drills are used for specific technical–tactical training as well as physical–physiological conditioning in combat sports. While the effects of different area sizes and number of within-round sparring partners on physiological and perceptive responses in combats sports were examined in previous studies, technical and tactical aspects were not investigated. This study investigated the effect of different within-round sparring partners number (i.e., at a time; 1 vs. 1, 1 vs. 2, and 1 vs. 4) and area sizes (2 m × 2 m, 4 m × 4 m, and 6 m × 6 m) variation on the technical–tactical aspects of small combat games in kickboxing.

**Method:**

Twenty male kickboxers (mean ± standard deviation, age: 20.3 ± 0.9 years), regularly competing in regional and national events randomly performed nine different kickboxing combats, lasting 2 min each. All combats were video recorded and analyzed using the software Dartfish.

**Results:**

Results showed that the total number of punches was significantly higher in 1 versus 4 compared with 1 versus 1 (*p* = 0.011, d = 0.83). Further, the total number of kicks was significantly higher in 1 versus 4 compared with 1 versus 1 and 1 versus 2 (*p* < 0.001; d = 0.99 and d = 0.83, respectively). Moreover, the total number of kick combinations was significantly higher in 1 versus 4 compared with 1 versus 1 and 1 versus 2 (*p* < 0.001; d = 1.05 and d = 0.95, respectively). The same outcome was significantly lower in 2 m × 2 m compared with 4 m × 4 m and 6 m × 6 m areas (*p* = 0.010 and d = − 0.45; *p* < 0.001 and d = − 0.6, respectively). The number of block-and-parry was significantly higher in 1 versus 4 compared with 1 versus 1 (*p* < 0.001, d = 1.45) and 1 versus 2 (*p* = 0.046, d = 0.61) and in 2 m × 2 m compared with 4 m × 4 m and 6 × 6 m areas (*p* < 0.001; d = 0.47 and d = 0.66, respectively). Backwards lean actions occurred more often in 2 m × 2 m compared with 4 m × 4 m (*p* = 0.009, d = 0.53) and 6 m × 6 m (*p* = 0.003, d = 0.60). However, the number of foot defenses was significantly lower in 2 m × 2 m compared with 6 m × 6 m (*p* < 0.001, d = 1.04) and 4 m × 4 m (*p* = 0.004, d = 0.63). Additionally, the number of clinches was significantly higher in 1 versus 1 compared with 1 versus 2 (*p* = 0.002, d = 0.7) and 1 versus 4 (*p* = 0.034, d = 0.45).

**Conclusions:**

This study provides practical insights into how to manipulate within-round sparring partners' number and/or area size to train specific kickboxing technical–tactical fundamentals.

***Trial registration*:**

This study does not report results related to health care interventions using human participants and therefore it was not prospectively registered.

## Background

Kickboxing is a striking combat sport, requiring complex skills and tactical excellence for success in competition [1]. A typical kickboxing match is composed of three 2-min rounds with 1-min rest in-between [2] and is disputed either in a ring (i.e., full-contact style) or in tatami styles (i.e., mats in light contact and point fighting [2]. During full-contact combat, the intention of the fighter is to beat his/her opponent using techniques delivered to legal targets with focus, speed, and determination [3]. The goal is to win the combat either by reaching a higher score or by executing a technical knockout [3]. In light contact kickboxing, competitors fight continuously using well-controlled techniques towards specific targets until the central referee commands "Stop" or "Break". Regarding point fighting style, the competition is similar to light contact in that kickboxers are required to use well-controlled techniques to legal targets to score points. However, upon each valid point, the combat is stopped by the central referee and the two judges to attribute the score [3]. For this, athletes use both upper (i.e., punching) and lower limb (i.e., kicking) techniques during offensive and defensive movements. The repetitive performance of these techniques requires high physical and physiological demands [1, 4]. As such, the main goal of kickboxing training is to prepare the kickboxers to effectively manage both the technical/tactical [3] and the physical/physiological [5, 6] demands of the combat.

To handle the competition demands, sparring drills are used by coaches as a specific modality for technical–tactical training as well as physical–physiological conditioning in combat sports [7]. Specifically, several studies in combat sports investigated the physiological aspects of small combat games (SCGs) by manipulating the effort-pause ratios [8, 9], the within-round sparring partners number [7, 10], and/or the area size [7–10]. In this sense, there is evidence indicating that using more than one opponent (i.e., 1 vs. 2 and 1 vs. 4 with sparring partners number changed every 1 min or 30 s during 2-min combat, respectively) is a good strategy to increase mean heart rate (HR) values (up to 90% of its maximal value) and rating of perceived exertion (RPE) in kickboxers [7]. The same effect on HR and RPE can be achieved by increasing high-intensity activities and decreasing low-intensity ones during simulated combat [7]. Regarding area size, it has been shown that restricting the combat area could affect time-motion variables in kickboxing. For instance, Ouergui et al. [7] reported a longer duration of high-intensity actions in the 2 m × 2 m area compared with the 4 m × 4 m and the 6 m × 6 m areas. However, studies dealing with the effects of different area sizes and number of within-round sparring partners on technical–tactical aspects of kickboxing are yet inexistent, highlighting a void in the literature. Indeed, the technical–tactical aspects are key for the athletes’ development and decisive for success in competition [10].

Therefore, this study aimed to investigate the technical–tactical aspects during the SCGs by manipulating the number of opponents (1 vs. 1, 1 vs. 2, and 1 vs. 4) and the area sizes (2 m × 2 m, 4 m × 4 m, and 6 m × 6 m). It was hypothesized that adding more than one within-round sparring partner and decreasing the area size would result in more frequent attacks and defensives techniques during bouts [7].

## Methods

### Participants

Twenty male kickboxers, belonging to the same club, volunteered to participate in this study (mean ± standard deviation, age: 20.3 ± 0.9 years; height: 177.0 ± 4.8 cm; body mass: 71.8 ± 10.5 kg). All participants regularly competed in intermediate (i.e., regional) and high-level (i.e., national) kickboxing events over the last 2 years. They have been training 3–5 days a week with 2 h per session. After a detailed explanation of the aim, benefits, and potential risks of the experimental procedures, written consent was gathered from all participants before taking part in this study. Athletes were also prescreened for injury before taking part in the present study. The study was conducted per the Declaration of Helsinki and approved by the local ethics committee.

### Experimental design

**Fig. 1:**
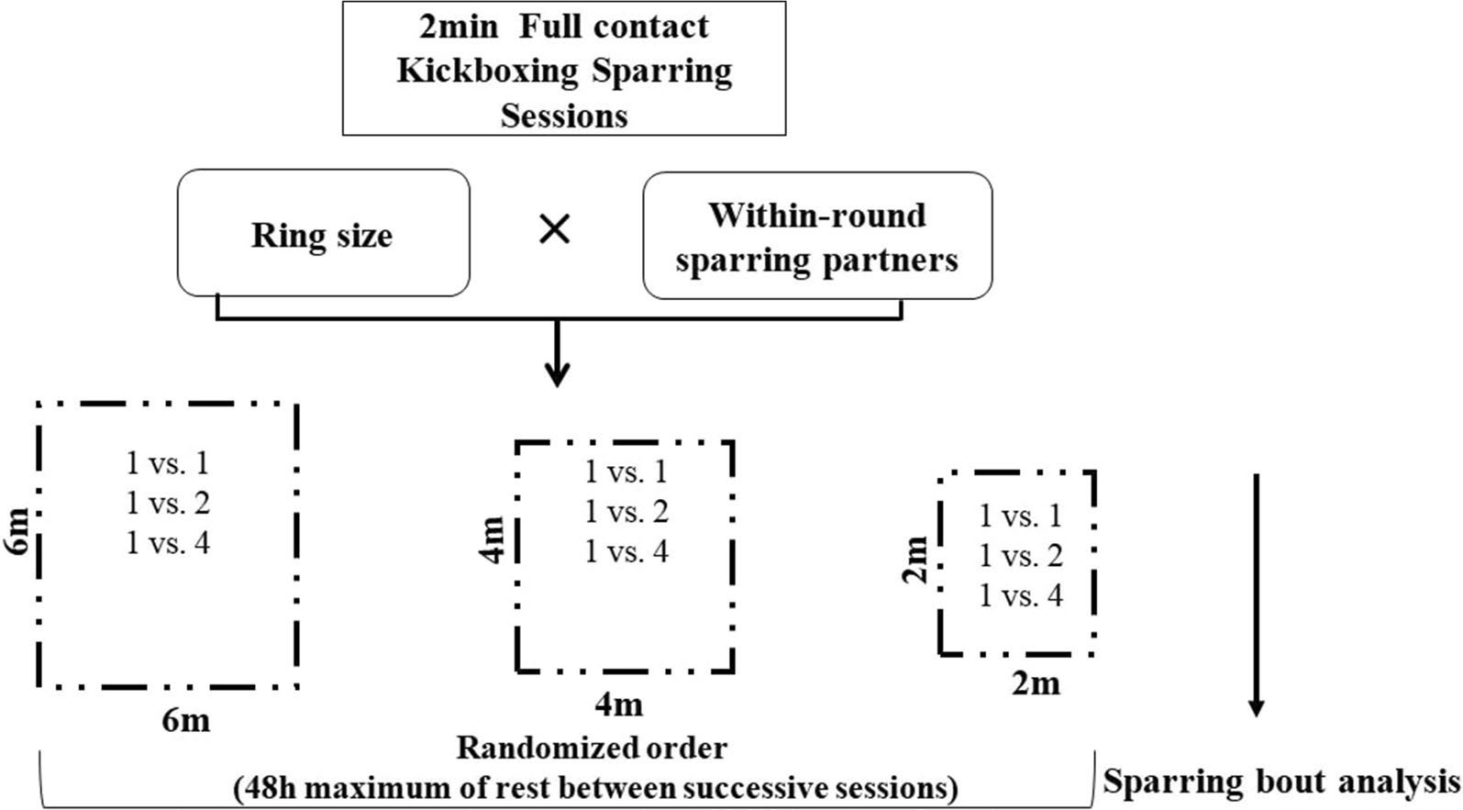
Schematic representation of the study design

In a randomized cross-over study design, kickboxers were exposed to nine different conditions resulting from combinations of different area sizes and numbers of within-round opponents as previously described by Ouergui et al. [7]. The experimentation consisted of performing sparring rounds of 2 min against different opponents (i.e., 1 vs. 1, 1 vs. 2, and 1 vs. 4) within a varied area size (i.e., 2 m × 2 m [small], 4 m × 4 m [medium] and 6 m × 6 m [large]) (Fig. 1). Specifically, whereas in the 1 versus 1 condition both kickboxers completed the whole sparring session, during the 1 versus 2 and 1 versus 4 conditions the opponent changed every 1 min or every 30 s, respectively [7]. The number of within-round sparring partners was chosen to let the kickboxers combating for a reasonable time, which could not be less than 30 s with every partner, resembling a real kickboxing match [7]. In terms of area, different formats were chosen to induce variation in combat distances as commonly used by coaches [7].

### Procedures

All kickboxers participated in a familiarization session 1 week before the beginning of the experimentation to be acquainted with the different conditions. Additionally, all sessions were conducted at the same time of day to avoid any risk of bias due to diurnal variation. Before each testing session, athletes performed 15 min of a standardized warm-up consisting of jogging and dynamic stretching followed by 3 min of passive rest. All sparring rounds were directed by two investigators (one is a qualified kickboxing coach) ensuring the safety of the kickboxers and providing verbal encouragement. Kickboxers were instructed to wear their official equipment for competition (i.e., helmet, mouth guards, gloves, roll bandages, shin guards, and foot protectors) during all sparring sessions. All bouts were recorded for subsequent analysis using 2 cameras (Canon 650D 18 Megapixels, ISO: 400, shutter speed: 1/125 s, f/4; Canon, Inc., Tokyo, Japan) placed at 1.5 m from the combat area. The experimental conditions were conducted in the pre-season period across 30 days with a minimum duration of 48 h but no more than 72 h between two subsequent sessions.

### Sparring-bouts analysis

The recorded video footages were analyzed frame by frame (interval = 0.016 s) using the Dartfish software (Dartfish Edition MPT34M Pro 5.5, Lausanne, Switzerland). The technical–tactical analysis included the determination of the offensive behavior represented by the total number of punches, kicks, punches combinations, kicks combinations, and punches and kicks combination. The defensive behavior was assessed by counting the total numbers of block-and-parry, slip, backwards leans, clinches and the duck and push actions. This method has been deemed reliable according to a previous study [3].

### Statistical analyses

Data are presented as mean and standard deviation. Sphericity was tested using the Mauchly test and a Greenhouse–Geisser correction was used when necessary (i.e., for kick combinations, slip and backwards leans variables) [11]. A two-way (number of opponents × area sizes) analysis of variance for paired data was used to compare the number of different technical–tactical actions across conditions and partial eta squared (η_p_^2^) effect size values were reported and classified as 0.01 = small, 0.09 = medium, 0.25 = large [12]. A Bonferroni test was used as post-hoc. Standardized effect size (Cohen *d*) analysis was used to interpret the magnitude of differences between conditions and classified according to Hopkins [13] *d* ≤ 0.2—trivial, 0.2 < *d* ≤ 0.6—small, 0.6 < *d* ≤ 1.2—moderate, 1.2 < *d* ≤ 2.0—large, 2.0 < *d* ≤ 4.0—very large and *d* > 4.0—extremely large. Moreover, upper and lower 95% confidence intervals of the difference (95%CIdiff) were calculated for the corresponding variation. The significance level was set a priori at 5%. All statistical calculations were performed using the statistical software package SPSS (IBM, version 23, IBM Corp., Armonk, NY, USA).

## Results

Table 1 presents the total number of punches and kicks during kickboxing SCGs executed with varied numbers of opponents and area sizes. The number of punches was only affected by the number of opponents (*F*_2,57_ = 4.69, *p* = 0.013, η_p_^2^ = 0.141) with a higher number observed in 1 versus 4 compared with 1 versus 1 condition (95%CIdiff = 1.42,14.01; *d* = 0.83; *p* = 0.011). Similarly, the number of kicks was only affected by number of opponents (*F*_2,57_ = 14.08, *p* < 0.001, η_p_^2^ = 0.331) with a higher number observed in 1 versus 4 compared with 1 versus 1 (95%CIdiff = 2.09,7.40; *d* = 0.99; *p* < 0.001) and 1 versus 2 (95%CIdiff = 2.46,7.8; *d* = 0.83; *p* < 0.001) conditions.

**Table 1:** Total numbers of punches and kicks during kickboxing small combat games with varied numbers of opponents and area sizes (values are mean ± standard deviation, n = 20)

	Total number of punches	Total number of kicks
	*1 versus 1*	
2 m × 2 m	33 ± 10	11 ± 3
4 m × 4 m	30 ± 9	11 ± 4
6 m × 6 m	31 ± 7	1 ± 2
	*1 versus 2*	
2 m × 2 m	36 ± 11	10 ± 7
4 m × 4 m	34 ± 13	11 ± 5
6 m × 6 m	33 ± 7	11 ± 6
	*1 versus 4*	
2 m × 2 m	38 ± 11^a^	16 ± 7^ab^
4 m × 4 m	40 ± 10^a^	14 ± 6^ab^
6 m × 6 m	39 ± 10^a^	17 ± 7^b^

^a^Main effect of number of opponents, different from 1 versus 1 (*p* < 0.01)

^b^Main effect of number of opponents, different from 1 versus 2 (*p* < 0.001)

Table 2 presents the total number of technique combinations during kickboxing SCGs executed with varied numbers of opponents and area sizes. Regarding kicks, there was a main effect of opponent (*F*_1,57_ = 12.14, *p* = 0.001, η_p_^2^ = 0.300) with higher number of combinations following 1 versus 4 condition compared with 1 versus 1 (95%CIdiff = 1.07,3.86; *d* = 1.05; *p* < 0.001) and 1 versus 2 (95%CIdiff = 0.97,3.76; *d* = 0.95; *p* < 0.001) conditions. There was also a main effect of area (*F*_1.497,85.326_ = 9.65, *p* = 0.001, η_p_^2^ = 0.145) with lower number of combinations for 2 m × 2 m compared with 4 m × 4 m (95%CIdiff = − 1.91, − 0.19; *d* = − 0.45; *p* = 0.010) and 6 m × 6 m (95%CIdiff = − 2.38, − 0.66; *d* = − 0.6; *p* < 0.001) areas. Additionally, results showed a significant opponent by area interaction (*F*_2.994,85.326_ = 3.85, *p* = 0.012, η_p_^2^ = 0.119) with the 1 versus 4 in 4 m × 4 m area resulting in higher number of kick combinations compared with 1 versus 1 in 2 m × 2 m (95%CIdiff = 0.74,5.76; *d* = 1.41; *p* = 0.001), 4 m × 4 m (95%CIdiff = 0.14,5.16; *d* = 1.014; *p* = 0.022), and 6 m × 6 m (95%CIdiff = 0.04,5.06; *d* = 1.01; *p* = 0.034) areas. The 1 versus 4 in 4 m × 4 m area showed a higher number of kick combinations than the 1 versus 2 in the 2 m × 2 m (95%CIdiff = 0.44,5.46; *d* = 1.17; *p* = 0.005), 4 m × 4 m (95%CIdiff = 0.14,5.16; *d* = 0.99; *p* = 0.022) and 6 m × 6 m (95%CIdiff = 0.04,5.06; *d* = 0.97; *p* = 0.034) areas, and the 1 versus 4 in 2 m × 2 m area (95%CIdiff = 0.24,4.26; *d* = 0.65; *p* = 0.013). Additionally, except 1 versus 4 in 4 m × 4 m area (*p* = 1.0), 1 versus 4 in the 6 m × 6 m resulted in higher number of kick combinations (*p* < 0.001 for all comparisons) compared with 1 versus 1 in 2 m × 2 m (95%CIdiff = 1.94,6.96; *d* = 1.8), 4 m × 4 m (95%CIdiff = 1.34,6.36; *d* = 1.38) and 6 m × 6 m (95%CIdiff = 1.24,6.26; *d* = 1.39) areas. The 1 versus 4 in the 6 m × 6 m showed a higher number of kick combinations than the 1 versus 2 in the 2 m × 2 m (95%CIdiff = 1.64,6.66; *d* = 1.53), 4 m × 4 m (95%CIdiff = 1.34,6.36; *d* = 1.35) and 6 m × 6 m (95%CIdiff = 1.24,6.26; *d* = 1.33); and then the 1 versus 4 in the 2 m × 2 m (95%CIdiff = 1.44,5.46; *d* = 0.94).

**Table 2:** Techniques combinations during kickboxing small combat games with varied numbers of opponents and area sizes (values are mean ± standard deviation, n = 20)

	Punches combinations	Punches and kicks combinations	Kicks combinations
		*1 versus 1*	
2 m × 2 m	7 ± 4	3 ± 3	0 ± 1^c,d,e^
4 m × 4 m	6 ± 3	4 ± 3	1 ± 1^d,e^
6 m × 6 m	7 ± 3	4 ± 3	1 ± 1^d,e^
		*1 versus 2*	
2 m × 2 m	5 ± 4	5 ± 4	1 ± 1^b,c,d,e^
4 m × 4 m	4 ± 4	4 ± 3	1 ± 1^d,e^
6 m × 6 m	5 ± 3	4 ± 3	1 ± 1^d,e^
		*1 versus 4*	
2 m × 2 m	5 ± 4	3 ± 3	1 ± 3^a,b,c,d,e^
4 m × 4 m	5 ± 3	6 ± 5	3 ± 4^a^
6 m × 6 m	5 ± 4	7 ± 6	5 ± 4^a^

^a^Main effect of number of opponent, different from 1 versus 1 and 1 versus 2 (*p* < 0.001)

^b^Main effect of area, lower in 2 m × 2 m compared with 4 × 4 m (*p* = 0.010)

^c^Main effect of area, lower in 2 m × 2 m compared with 6 m × 6 m (*p* < 0.001)

^d^Number of opponents and area interaction effect, lower than 1 versus 4 in 4 m × 4 m (*p* < 0.05)

^e^Number of opponent and area interaction effect, lower than 1 versus 4 in 6 m × 6 m (*p* < 0.001)

Table 3 presents the defensive actions during kickboxing SCGs executed with varied numbers of opponents and area sizes. Regarding block-and-parry, there was a main effect of opponents number (*F*_2,57_ = 15.03, *p* < 0.001, η_p_^2^ = 0.345) with higher number for 1 versus 4 compared with 1 versus 1 (95%CIdiff = 4.71,2.43; *d* = 1.45; *p* < 0.001) and 1 versus 2 (95%CIdiff = 0.06,7.78; *d* = 0.61; *p* = 0.046). Additionally, the number of block-and-parry was higher for 1 versus 2 compared with 1 versus 1 (95%CIdiff = 0.79,8.51; *d* = 0.85; *p* = 0.013). There was also a main effect of area (*F*_2,114_ = 20.56, *p* < 0.001, η_p_^2^ = 0.265) with higher number of block-and-parry in 2 m × 2 m compared with 4 m × 4 m (95%CIdiff = 1.64,5.5; *d* = 0.47; *p* < 0.001), and 6 m × 6 m (95%CIdiff = 3.00,6.84; *d* = 0.66; *p* < 0.001) areas. Additionally, there was a significant opponent by area interaction (*F*_4,114_ = 8.36, *p* < 0.001, η_p_^2^ = 0.227) with 1 versus 2 in 2 m × 2 m area resulting in higher number of block-and-parry compared with 1 versus 1 in 2 m × 2 m (95%CIdiff = 2.79,15.61; *d* = 1.57; *p* < 0.001), 4 m × 4 m (95%CIdiff = 2.84,15.66; *d* = 1.79; *p* < 0.001) and 6 m × 6 m (95%CIdiff = 1.29,14.11; *d* = 1.22; *p* = 0.004) areas. The 1 versus 2 in 2 m × 2 m area showed a higher number of block-and-parry than the 1 versus 2 in 4 m × 4 m (95%CIdiff = 0.2,9.2; *d* = 0.71; *p* = 0.031) and 6 m × 6 m (95%CIdiff = 3.00,12.00; *d* = 1.31; *p* < 0.001) areas. The 1 versus 4 in 4 m × 4 m area resulted in higher number of block-and-parry compared with 1 versus 1 in 2 m × 2 m (95%CIdiff = 1.59,14.41; *d* = 1.47; *p* = 0.002), 4 m × 4 m (95%CIdiff = 1.64,14.46; *d* = 1.68; *p* = 0.002) and 6 m × 6 m (95%CIdiff = 0.09,12.91; *d* = 1.1; *p* = 0.036) areas. Additionally, 1 versus 4 in 2 m × 2 m resulted in higher number of block-and-parry compared with 1 versus 1 in 2 m × 2 m (95%CIdiff = 7.54,20.36; *d* = 2.05; *p* < 0.001), 4 m × 4 m (95%CIdiff = 7.59,20.41; *d* = 2.28; *p* < 0.001), and 6 m × 6 m (95%CIdiff = 6.04,18.86; *d* = 1.71; *p* < 0.001) areas. The 1 versus 4 in 2 m × 2 m showed a higher number of block-and-parry than the 1 versus 2 in 4 m × 4 m (95%CIdiff = 3.04,15.86; *d* = 1.25; *p* = 0.001) and 6 m × 6 m (95%CIdiff = 5.84,18.66; *d* = 1.82; *p* < 0.001) areas; and than the 1 versus 4 in 4 m × 4 m (95%CIdiff = 1.45,10.45; *d* = 0.83; *p* = 0.001) and 6 m × 6 m (95%CIdiff = 4.25,13.25; *d* = 1.18; *p* < 0.001) areas.

**Table 3:** Defensives actions during kickboxing small combat games executed with varied numbers of opponents and area sizes (values are mean ± standard deviation; n = 20)

	Block/Parry	Slip	Lean backward	Foot defense	Duck	Clinches	Push
	*1 versus 1*						
2 m × 2 m	14 ± 5^d,e,f,g^	1 ± 1	1 ± 1^i^	1 ± 1	0.2 ± 0.4	1 ± 2	1 ± 1^¡^
4 m × 4 m	14 ± 4^e,f,g^	2 ± 3	0 ± 0	2 ± 2^ l^	0.4 ± 0.6	1 ± 2	0 ± 1^¡^
6 m × 6 m	15 ± 6^e,f,g^	2 ± 3	0 ± 1	2 ± 2^j,k^	0.3 ± 0.6	1 ± 1	0 ± 1^¡^
	*1 versus 2*						
2 m × 2 m	23 ± 7^c,d^	1 ± 2	1 ± 1^i^	1 ± 1	0 ± 0	1 ± 1	1 ± 1
4 m × 4 m	18 ± 7^c,e,g^	1 ± 1	0 ± 1	1 ± 1^ l^	0 ± 1	0 ± 1	0 ± 1
6 m × 6 m	15 ± 5^c,e,g^	2 ± 2	0 ± 1	2 ± 2^j,k^	0 ± 0	0 ± 0	0 ± 1
	*1 versus 4*						
2 m × 2 m	28 ± 8^a,b,d^	4 ± 4	1 ± 1^i^	1 ± 1	0 ± 1	0 ± 1	1 ± 1
4 m × 4 m	22 ± 6^a,b,g^	2 ± 2	0 ± 1	1 ± 1^ l^	0 ± 0	1 ± 1	0 ± 1
6 m × 6 m	19 ± 6^a,b,g^	2 ± 2	0 ± 1	2 ± 2^j,k^	0 ± 0	1 ± 1	0 ± 1

^a^Number of opponents main effect, different from 1 versus 1 (*p* < 0.001)

^b^Number of opponent main effect, different from 1 versus 2 (*p* < 0.05)

^c^Number of opponents main effect, different from 1 versus 1 (*p* < 0.05)

^d^Main effect of area, different from 4 m × 4 m and 6 m × 6 m (*p* < 0.001)

^e^Number of opponents and area interaction effect, lower than 1 versus 2 in 2 m × 2 m (*p* < 0.05)

^f^Number of opponents and area interaction effect, lower than 1 versus 4 in 4 m × 4 m (*p* < 0.05)

^g^Number of opponents and area interaction effect, lower than 1 versus 4 in 2 m × 2 m (*p* < 0.01); i = main effect of area, different from 4 m × 4 m and 6 m × 6 m (*p* < 0.05); j = a main effect of area, different from 2 m × 2 m (*p* < 0.001); k = main effect of area, different from 4 m × 4 m (*p* < 0.05); l = main effect of area, different from 2 m × 2 m (*p* < 0.01)

^¡^Number of opponents main effect, different from 1 versus 2 and 1 versus 4 conditions (*p* < 0.05)

With respect to slips, there was a significant opponent by area interaction (*F*_3.308,94.267_ = 4.53, *p* = 0.004, η_p_^2^ = 0.137), but the Bonferroni test indicated only a tendency towards a significant difference between 1 versus 1 in 2 m × 2 m and 1 versus 4 in 2 m × 2 m area (95%CIdiff = − 4.97,0.17; *d* = 0.91; *p* = 0.083), and between 1 versus 4 in 2 m × 2 m area and 1 versus 4 in 4 m × 4 m area (95%CIdiff = − 0.05,4.05; *d* = 0.76; *p* = 0.064).

Regarding lean backwards, there was a significant effect of area (*F*_1.600,91.220_ = 6.86, *p* = 0.003) with a higher number for 2 m × 2 m compared with 4 m × 4 m (95%CIdiff = 0.08,0.78; *d* = 0.53; *p* = 0.009), and 6 m × 6 m (95%CIdiff = 0.13,0.83; *d* = 0.60; *p* = 0.003) areas. Regarding foot defenses, a significant effect of area was detected (*F*_2,114_ = 16.45, *p* < 0.001, η_p_^2^ = 0.224) with higher number for 6 m × 6 m compared with 2 m × 2 m (95%CIdiff = 0.83,2.07; *d* = 1.04; *p* < 0.001), and 4 m × 4 m (95%CIdiff = 0,1.23; *d* = 0.37; *p* = 0.050) areas. Likewise, the number of foot defenses was significantly higher for 4 m × 4 m compared with 2 m × 2 m area (95%CIdif = 0.22,1.45; *d* = 0.63; *p* = 0.004).

In terms of clinches, a significant effect of the factor opponent was noted (*F*_2,57_ = 6.84, *p* = 0.002, η_p_^2^ = 0.194) with a higher number for 1 versus 1 compared with 1 versus 2 (95%CIdiff = 0.21,1.16; *d* = 0.7; *p* = 0.002) and 1 versus 4 (95%CIdiff = 0.03,0.97; *d* = 0.45; *p* = 0.034) conditions.

## Discussion

This study examined the effects of within-round sparring partners number and area size on the technical and tactical aspects during SCGs in kickboxing. The main findings showed that the 1 versus 4 condition imposes a higher number of both offensives (i.e., punches, kicks, and kick combination) and defensives actions (i.e., block-and-parry actions) compared with the 1 versus 1 condition. Regarding area sizes, 2 m × 2 m affords a higher number of lean backward as well as block-and-parry actions compared with 4 m × 4 m and 6 m × 6 m areas. However, the number of kicks combination and foot defenses were higher in the 4 m × 4 m and 6 m × 6 m areas compared with the 2 m × 2 m area.

The present study showed that when a kickboxer was confronted by more than one opponent (i.e., 1 vs. 2 or 1 vs. 4), offensive and defensive techniques occur more frequently. More specifically, our findings showed that changing within-round sparring partners' number (at a time) increased the rate of punching and kicking. Such an observation could be explained by the fact that the new sparring partner contributes to maintain and/or increase the cadence (therefore the effort-pause ratio, as well) of the sparring simply because of his fresh physical state [7]. An earlier study demonstrated that changing opponents during SCGs increases the duration of high-intensity actions in comparison with the condition when opponents do not change [7].

Since the execution of kicks does not need to get close to the opponent [14], these techniques seem to be used as a tactical way to maintain a favorable distance with the opponents to execute more high-intensities techniques. Moreover, to create uncertainty for the opponent by achieving different legal targets, athletes used combinations such as kicks combinations [3]. The reason for the increased number of punches is that these techniques’ type is so effective and requires a low amount of energy to be operated, as already shown in (1 vs. 1) in boxing matches [15]. These findings are supported by previous research results in kickboxing [3, 16]. These authors have shown that jab-cross punches and roundhouse kicks are the most effective techniques to win the fight. Specifically, Ambrozy et al. [16] showed that high roundhouse kick was the most effective lower limb technique which often lead to an end of the fight with a knock-out.

As a general rule, to win a kickboxing match, kickboxers should deliver more offensive techniques alongside effective defensive skills [3]. In the case when a kickboxer was confronted by new opponent, more defensive techniques were used than in the 1 versus 1 condition. This seems to be a strategy to cope with fatigue given that attack techniques are metabolically demanding [7]. Moreover, the new opponent tended to adopt an offensive strategy forcing his rival to rely on defensive actions in order not to be hit with a knockdown or knockout punch. In the present study, block-and-parry actions were the defensive skills most used to avoid sparring opponents’ attacks especially during the 1 versus 4 condition. Our results are partially supported by previous studies [3, 15], which showed that these defensive techniques were among the most frequent arm skills used in amateur boxing and kickboxing. On the other hand, clinches were more frequently used during the 1 versus 1 than during the 1 versus 4 condition. Similar to other striking combat sports (i.e., amateur boxing), it seems that the increase in clinching movements during the 1 versus 1 condition was used as a pacing strategy to avoid fatigue throughout bouts [17]. However, the findings of our study indicated that clinching was not the preferred option to draw a stoppage and gain a brief rest when the kickboxer was confronted by four different opponents (i.e., 1 vs. 4). It can be hypothesized that, due to its both upper and lower limb-combat nature, kickboxing is featured by more clinching’s use only when both contenders are similarly fatigued.

The distance between opponents is an important factor that influences the technical–tactical behavior of combat sports athletes, including kickboxing [15]. In the current study, it seemed that technique occurrence was driven by the combat distance. Namely, when the combat area was restricted, kickboxers used more defensive techniques with the number of backward lean and block-and-parry being higher in the 2 m × 2 m area compared with the 4 m × 4 and 6 m × 6 m areas. These techniques are generally used to prevent or avoid the opponent’s punches [3]. In amateur boxing, it was found that the most effective and frequent defensive skill used the trunk (i.e., lean backward) as it requires a complete mastery of technique and obliges the boxer to employ it before initiating any counter-attacks [15]. Thus, reducing the area size forced kickboxers to keep a close distance. Such a close distance resulted in kickboxers delivering more punches [7]. Therefore, since the number of offensive actions delivered to the tested athlete increased, the later reacted by increasing the number of his defensive actions. In this context, Ouergui et al. [7] suggested that during SCGs—a proxy for regular combats—athletes need to improve their technical and tactical skills in reaction to the opponents’ techniques. It seems also plausible to argue that the increased usage of defensive skills was not only for tactical ends in response to the opponent's actions but also as a strategy to pace effort and minimize fatigue. In this sense, Dunn et al. [17] revealed that amateur boxers reduced the total number of attacks and increased guard drops as part of a pacing strategy approach to avoid fatigue throughout the bout. In this sense, Rydzik and Ambroży [4] showed that the high level of physical fitness underlies the optimal development of technique for kickboxing competitors.

Our findings indicated that the number of kicks combinations and foot defenses decreased with the restriction of the area size. In this context, Slimani et al. [18] reported that in general, lower limb techniques and kicks combinations (i.e., low-kick-roundhouse kick) were not predominant in low-kick kickboxing competitions. This could be related to the long trajectory as well as the long time it takes kicking techniques to be performed [13, 18]. As such, kicks combinations and foot defenses appear not to be the best skills to use when the area size is reduced. This can be explained by the fact that the 6 m × 6 m area allows more space for stepping actions than in the 2 m × 2 m and therefore it is possible to keep a favorable distance from the opponent to execute such kicking techniques [7]. In fact, fighting in a 6 m × 6 m area allows kickboxers to better perceive how to reach the target by finding the optimal attack distance. Contrarily, in the 2 m × 2 m condition, the contenders are too close to each other to allow for the preparatory actions to execute high-intensity kick techniques properly [7]. Consequently, the 2 m × 2 m area size is not a favorable condition to initiate counter-attacks (e.g., the use of foot defense to escape) and therefore it is appropriate to limit the appearance of such offensive behaviors (i.e., kicks combinations) within it. In practice, this can be a goal that coaches might set to favor a particular tactical picture.

This study is not without limitations, which we would like to acknowledge. First, some morphological characteristics (i.e., weight categories and limbs lengths) were not considered as possible moderators of the effects of area size and different within-round sparring partners numbers on the technical–tactical skills of kickboxers. Moreover, the effort-pause ratio was not taken into account, which could provide more explanations regarding the technical–tactical aspects investigated during the different SCGs structures considered in the present study. Future studies should take into consideration these variables to better understand the effect of sparring drills on the technical–tactical aspects of kickboxers.

## Conclusions

The main findings of this study indicated that offensive (i.e., punches, kicks, and kicks combinations) and defensive (i.e., block-and-parry) actions occurred more frequently during the 1 versus 4 compared with the 1 versus 1 condition. These findings can be used by coaches to better design training sessions aimed at developing athletes’ technical–tactical skills. More specifically, coaches, who would like to emphasize the development of offensive/defensive drills, should favor the 1 versus 4 over the 1 versus 1 condition. On the other hand, the 1 versus 1 condition can be chosen by the coach to emphasize clinches as it affords a higher number of this specific action than the 1 versus 4 condition. In addition, a restricted area size (i.e., 2 m × 2 m) can be used to develop defensive techniques such as block-and-parry and lean backward. Differently, the 4 m × 4 m and the 6 m × 6 m areas seem to be more favorable to develop kicks combinations and foot defenses. Overall, for a successful performance in kickboxing, training strategies must be directed toward the development and improvement of both offensive and defensive actions. In fact, coaches are warmly advised to use SCGs to improve desired technical and tactical skills of kickboxers by using the adequate combination between areas and within-round sparring partners’ number. Finally, it would be relevant relating the outcomes of the SCGs with those during an ecologically valid environment (i.e., official match). This should be addressed in future studies.

## Data Availability

The datasets generated and/or analysed during the current study are not publicly available. Upon request, the corresponding author will share the data set.

## Abbreviations

SCGs: Small combat games
HR: Heart rate
RPE: Rating of perceived exertion
95%CIdiff: 95% Confidence intervals of the difference
